# Clinical Perspective of 3D Total Body Photography for Early Detection and Screening of Melanoma

**DOI:** 10.3389/fmed.2018.00152

**Published:** 2018-05-23

**Authors:** Jenna E. Rayner, Antonia M. Laino, Kaitlin L. Nufer, Laura Adams, Anthony P Raphael, Scott W Menzies, H. Peter Soyer

**Affiliations:** ^1^Dermatology Research Centre, The University of Queensland, The University of Queensland Diamantina Institute, Brisbane, QLD, Australia; ^2^Dermatology Department, Princess Alexandra Hospital, Brisbane, QLD, Australia; ^3^Sydney Melanoma Diagnostic Centre, Royal Prince Alfred Hospital, Camperdown, NSW, Australia; ^4^Sydney Medical School, The University of Sydney, Sydney, NSW, Australia

**Keywords:** melanoma, prevention, early detection, total body photography, dermoscopy, skin cancer

## Abstract

Melanoma incidence continues to increase across many populations globally and there is significant mortality associated with advanced disease. However, if detected early, patients have a very promising prognosis. The methods that have been utilized for early detection include clinician and patient skin examinations, dermoscopy (static and sequential imaging), and total body photography via 2D imaging. Total body photography has recently witnessed an evolution from 2D imaging with the ability to now create a 3D representation of the patient linked with dermoscopy images of individual lesions. 3D total body photography is a particularly beneficial screening tool for patients at high risk due to their personal or family history or those with multiple dysplastic naevi—the latter can make monitoring especially difficult without the assistance of technology. In this perspective, we discuss clinical examples utilizing 3D total body photography, associated advantages and limitations, and future directions of the technology. The optimal system for melanoma screening should improve diagnostic accuracy, be time and cost efficient, and accessible to patients across all demographic and socioeconomic groups. 3D total body photography has the potential to address these criteria and, most importantly, optimize crucial early detection.

## Melanoma incidence and screening

The incidence of invasive melanoma is increasing in the majority of Caucasian populations across the globe, including in people under 45 years of age (1). Australia and New Zealand are an interesting anomaly, where after an intensive 30-year sun education campaign, the incidence of invasive melanoma is decreasing in those under the age of 60 (2, 3). Despite this, Australia is experiencing a rapid rise in the incidence of *in situ* melanomas across all ages (3) and the state of Queensland has the highest incidence of melanoma in the world (4, 5). Melanoma is a significant and persisting global disease.

There are many factors influencing melanoma incidence and statistics. It is established that survival from melanoma is strongly correlated with tumor thickness at diagnosis. Thin melanomas (<0.8 mm) have a 10-year survival approaching 98%, in comparison to melanomas >4 mm, which at best have approximately 75% survival at 10 years (6).

Delayed melanoma detection also has a significant financial impact. Advanced melanoma cost an estimated AU$422 million nationally in 2014, with direct health costs making up 39% of that figure (7). Early detection of melanoma is critical to reducing the physical, emotional, and economic burden.

Despite these confronting statistics, the mortality benefit from skin cancer screening via full skin examinations has been difficult to establish due to a lack of available data (8). If a reliable, accessible, and cost-effective melanoma screening technology was available, there could be clearer guidelines for skin cancer screening, similar to those for cervical, bowel and breast cancer. This would significantly benefit high risk patients, such as those with a personal or family history of melanoma or multiple dysplastic naevi.

## Photographic imaging in dermatology

Dermatology is a highly visual speciality which can be greatly aided by photographic documentation. Images may be used to monitor response to treatment, document the location of lesions biopsied, and monitor disease progression. In monitoring pigmented lesions, dermatologists have traditionally been dependent on visual assessment, clinical memory recall and, if available, a 2D digital camera. However, the manipulation of a 3D surface such as the human skin into a 2D photograph can compromise the accuracy of the image. Composing a body map of a patient via 2D imaging is also time consuming. It requires multiple separate images of the patient to be taken in a variety of anatomical positions which may then overlap or conversely fail to include naevi if they are not captured from the specific anatomical angle of the single camera.

Despite the disadvantages of 2D imaging, it is still a broadly utilized and popular approach to total body photography. A study in the US in 2002 found that total body photography was recommended by clinicians at 87% of dermatology institutions to manage patients with >5 dysplastic naevi (9).

2D total body photography and sequential digital dermatoscopic imaging (SDDI) have been independent tools for monitoring pigmented naevi, utilized by dermatologists and skin cancer doctors (10, 11). Combining these resources optimizes the benefits of each technology and avoids the limitations of their singular use.

## Advances in dermatological imaging

Total body photography has recently witnessed an evolution from 2D imaging, with the ability to now create a 3D representation of the patient. A 3D imaging system combining total body photography and SDDI of individual lesions allows all existing melanocytic lesions on a patient to be monitored from a macroscopic perspective in combination with the dermoscopy morphology. Dermoscopy has been shown to significantly improve the sensitivity in the diagnosis of melanoma in multiple clinical studies (12). These findings were consistent with previous meta-analyses comparing dermoscopy to unassisted visual inspection (13, 14).

Since 2015, a prototype 3D total body photography imaging system (Vectra WB360, Canfield Scientific Inc, Parsippany, NJ, USA) composed of 46 cameras, has been used in clinical trials for monitoring high risk individuals at the Princess Alexandra Hospital, Brisbane, Australia. In 2017, the commercial system was launched, with improved imaging capabilities via 92 cameras and white or cross-polarized lighting. The cameras capture the images simultaneously and then construct a digital 3D avatar of the patient. The patient is required to hold only one anatomical position and the image capture happens within a few seconds. The 3D representation facilitates 360-degree rotation to view all body angles, including curved surfaces which are particularly compromised on 2D imaging. Although the 3D image consists of a relatively high-resolution macroscopic image, additional digital dermoscopy, which captures an image similar to what is seen through a handheld dermatoscope, is often required for complete assessment. The dermoscopy images are linked to the corresponding lesion marked on the 3D avatar. This ensures accurate documentation of the exact anatomical location of each lesion, in order to assess evolution over time. Serial imaging sessions consisting of total body and dermoscopy data can be reviewed by the treating dermatologist.

## Advantages of 3D total body photography: potential to improve the benign to malignant ratio

One risk of screening for early melanoma detection is the risk of high benign-to-malignant excision ratios. Although required for histopathology, biopsies have several limitations including potential adverse cosmetic and functional effects, cost, increased patient anxiety and decreased workplace productivity. Patient risk and anxiety and physician expertise influence the benign-to-malignant ratio (15), and the SCREEN study in Schleswig-Holstein, Germany, found that 20 to 55 excisions were performed to diagnose one melanoma (16).

Studies have shown that 2D total body photography may reduce the number of naevus biopsies and improve diagnostic accuracy in high risk melanoma patients (15). The use of dermoscopy alone has been shown to reduce the benign-to-malignant ratio of excised melanocytic lesions and reduce the number of patients referred for biopsy (17–20). Furthermore, SDDI has also been shown to achieve these outcomes independently of static examination (19, 21), because change in naevi is one of the most sensitive ways to detect potential malignant transformation. 3D total body photography with sequential digital dermatoscopic imaging (SDDI) allows physicians to prove naevi stability and improve the benign-to-malignant ratio (22). The efficiency of dermoscopy as a diagnostic tool to monitor and diagnose pigmented lesions is optimized if these lesions can be captured and serially recorded in the context of a 3D body map. This benefit may be heightened in patients with large numbers of dysplastic naevi.

## Clinical example

**Figure 1:**
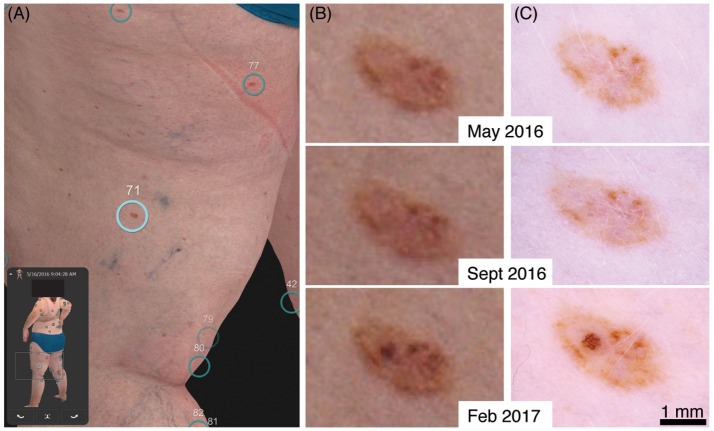
Evolution of a stable naevus to a melanoma *in situ*. **(A)** 3D total body map of naevi at first visit. **(B)** Digitally magnified 3D total body map of corresponding naevus at first, second and third visit. **(C)** Respective dermoscopy image at each visit.

A clinical example of early detection with our 3D total body photography and digital dermoscopy system can be seen in a 50-year-old female patient with a family and personal history of multiple melanoma (Figure 1 and Supplementary Figure 1). Her baseline imaging on visit 1 detected multiple naevi, including a rather benign-appearing naevus on her left posterior thigh (Figures 1A–C). At visit 2, 4 months later, the naevus was stable (Figures 1B,C middle panels). However at visit 3, 9 months from her original baseline imaging, a significant increase in pigment and asymmetrical broadened network was observed (Figures 1B,C bottom panels). She was referred to her GP for a diagnostic excision with 5 mm margins to exclude melanoma *in situ*. The histopathology report summarized that it was a “severely dysplastic compound naevus with features consistent with melanoma *in situ*.” Of note, the magnified body map images (Figure 1B) have reduced resolution in comparison to dermoscopy. However, in this case the same clinical decision could have been made on the magnified body map images alone. As the 3D imaging systems improve, the need for additional dermoscopy may be reduced.

## Advantages of 3D total body photography: early detection

The past decade has seen significant advances in the treatment of metastatic melanoma in what was previously a disease with limited treatment options and a dismal prognosis. However, despite continuing novel developments, metastatic melanoma treatments still have variable response rates and toxicities (23). Primary prevention and early detection (secondary prevention) of melanoma are still absolutely paramount.

3D total body photography and SDDI can play a significant role in the early detection of melanoma. The process of imaging also allows the patient to closely visualize their total skin surface in addition to individual lesion dermoscopy images on the screen. It is our observation that patients are often highly engaged in this process. It is possible that this technology could play a role in reinforcing the importance of ongoing preventative behaviors and self-skin checks, in addition to being a tool for the early detection of melanoma and non-melanoma skin cancers (24).

## Advantages of 3D total body photography: remote assessment of images for rural areas

3D total body photography and SDDI technology may be leveraged to increase service availability in remote and rural areas and reduce the financial impact of melanomas. Achieving these important outcomes is particularly challenging in a country such as Australia, given that a significant proportion of our high risk population lives in regional and rural communities with decreased access to health care services (25). A study using data from the Queensland Cancer Registry found the fatality rate for melanoma was 20% higher in rural versus urban areas (26). The age-standardized melanoma death rate in regional and remote areas has been reported as decreasing more slowly than in metropolitan areas (27).

A system such as the 3D total body photography and SDDI used at our hospital has the potential to be established at various remote sites and operated by nurses, clinical assistants and junior doctors, who can then send images to teledermatologists. Though these alternate specialist pathways may not seem ideal, they address the reality and challenges of vast geographical distance in a country like Australia. Dermatology telehealth services are already in use within Queensland Health, using electronic transfer of digital clinical and dermoscopy images. Given the additional data obtained from 3D imaging systems, we foresee their incorporation via PACS (Picture Archiving and Communication System) for review by dermatologists locally or off-site, with reporting into the integrated Electronic Medical Record. However, prior to introduction, the systems will need to achieve Digital Imaging and Communications in Medicine (DICOM) compliance, which is an ongoing initiative via the International Skin Imaging Collaboration (ISIC) (28, 29).

## Advantages of 3D total body photography: reduction in patient and physician anxiety

3D total body photography and SDDI can also reduce patient anxiety and assist practitioners in their management approach. In a study of more than 100 patients with a personal history of melanoma, their worry was reduced on all scales after undergoing conventional 2D total body photography (30).

There has been some concern amongst physicians that photographic documentation of pigmented lesions could potentially increase medical liability because of accusations of missed diagnoses of melanoma. However, two studies conducted in the US found no reports of medical malpractice resulting from the use of conventional total body photography (9, 31). In fact, total body photography could actually be of benefit in malpractice cases by documenting appropriateness of care.

## Limitations in 3D total body photography

The nature of medicine is never definitive and often complex. The majority of melanomas are pigmented and detection and monitoring can be assisted via technology such as 3D total body photography and SDDI systems. However, 2–8% of melanomas are hypopigmented (32) and detection on a 3D total body photography imaging system may be less reliable than with pigmented lesions. The role of dermatologists and skin cancer doctors is not replaced, but instead assisted, through 3D total body photography and SDDI. Current logistical limitations of the system include the large physical size of the unit and the significant expense and complexities around IT management and storage. 3D total body photography does not allow for monitoring of lesions in the genital, acral and scalp body sites nor within body folds. Dermoscopy images also require interpretation by a dermatologist familiar with dermoscopy. Studies have shown dermoscopy by untrained or less experienced individuals was no better than inspection with the naked eye (13).

Additionally, biopsy efficiency may be reduced in younger populations where benign naevi are still increasing in number and changing in morphology. In reviewing naevus biopsies in patients monitored by total body photography, Truong et al. found that higher biopsy rates occurred in younger patients (<30 years old), although it would appear these patients had total body photography alone and not combined with SDDI (15).

Lastly, even with the current resolution of magnified 3D bodymap images as shown in Figure 1B, the integration of SDDI is still recommended. As the technology improves and the need for additional SDDI is negated, the clinical utility of 3D total body photography will become evident, through improved lesion identification and tracking in combination with reduced appointment time and healthcare costs. However, 3D total body photography is still in its infancy, requiring ongoing research and validation before it will have a significant impact on standard dermatological care.

## Future directions

Future technologies for monitoring melanocytic naevi, including mobile smartphone applications, computer assisted diagnosis and genetic risk profiling, are likely to be integrated with total body photography (33). Smartphone technology is an evolving field of research in the detection of melanomas (34). A population-based survey of individuals with melanoma found that over half of all melanomas were self-detected (35), so mobile phone technology may have the potential to allow communication between patient dermatologists.

Another field of research that has received significant interest is the development of algorithms for the automatic analysis of melanocytic lesions. A recent landmark paper by Esteva and colleagues demonstrated for the first time artificial intelligence that classified both keratinocyte and melanoma skin cancer with a standard of competency that was comparable to dermatologists (36). With such advancements, it is very likely that artificial intelligence, first via machine-assistance and then full automation, will be a key technology in revolutionizing the clinical application of both 2D and 3D imaging in dermatology.

Ongoing research on the mortality benefit from skin cancer screening via full skin examinations, and in particular in regard to 3D total body photography and SDDI, would be a valuable contribution to the current clinical practice. Research comparing the efficacy of 2D and 3D imaging will help to justify the cost effectiveness of the technology, facilitate funding and eventually lead to clinical uptake. Our ultimate goal remains that “No one should die of malignant melanoma” as so eloquently set forward by AB Ackerman 30 years ago (37).

As evident from the incidence, emotional, and financial cost of melanoma, there is much to gain from improving the efficiency of early detection and reducing the burden of advanced melanoma. The ideal imaging system should increase the diagnostic accuracy of all lesions, be time and cost efficient, reduce benign biopsies, and be accessible to patients across all demographic and socioeconomic groups. The current 3D total body photography and sequential digital dermatoscopic imaging can potentially fulfill these criteria.

## Ethics statement

The study was approved by Metro South Health Human Research Ethics Committee (HREC) located at Princess Alexandra Hospital and The University of Queensland HREC in accordance with the National Statement on Ethical Conduct in Human Research (2007), the International Guidelines known as Good Clinical Practice and the World Medical Association's Declaration of Helsinki.

## Author contributions

JR wrote manuscript, conducted studies. AL, KN, and LA edited manuscript, conducted studies. AR edited and wrote manuscript, prepared figures, interpretation of data. SM and HS edited and wrote manuscript, interpretation of data.

### Conflict of interest statement

HS Shareholder of e-derm consult GmbH and MoleMap by Dermatologists Pty Ltd. He provides teledermatological reports regularly for both companies. AR and HS: consults for Canfield Scientific. The other authors declare that the research was conducted in the absence of any commercial or financial relationships that could be construed as a potential conflict of interest. The reviewer TS and handling Editor declared their shared affiliation.

## Supplementary material

Supplementary Figure 1 **(A)** The rings without pigmented lesions (#79, #80) alert that there are naevi on the medial aspect of the leg which are not visible on this lateral 2D screen shot **(B)**. In practice, the touch screen allows the body map to be rotated and the images viewed.
