# Supplementary figures and images for: Clinical Perspective of 3D Total Body Photography for Early Detection and Screening of Melanoma
Source: Front Med (Lausanne). 2018 May 23;5:152. doi: 10.3389/fmed.2018.00152 (PMC5992425; doi:10.3389/fmed.2018.00152)

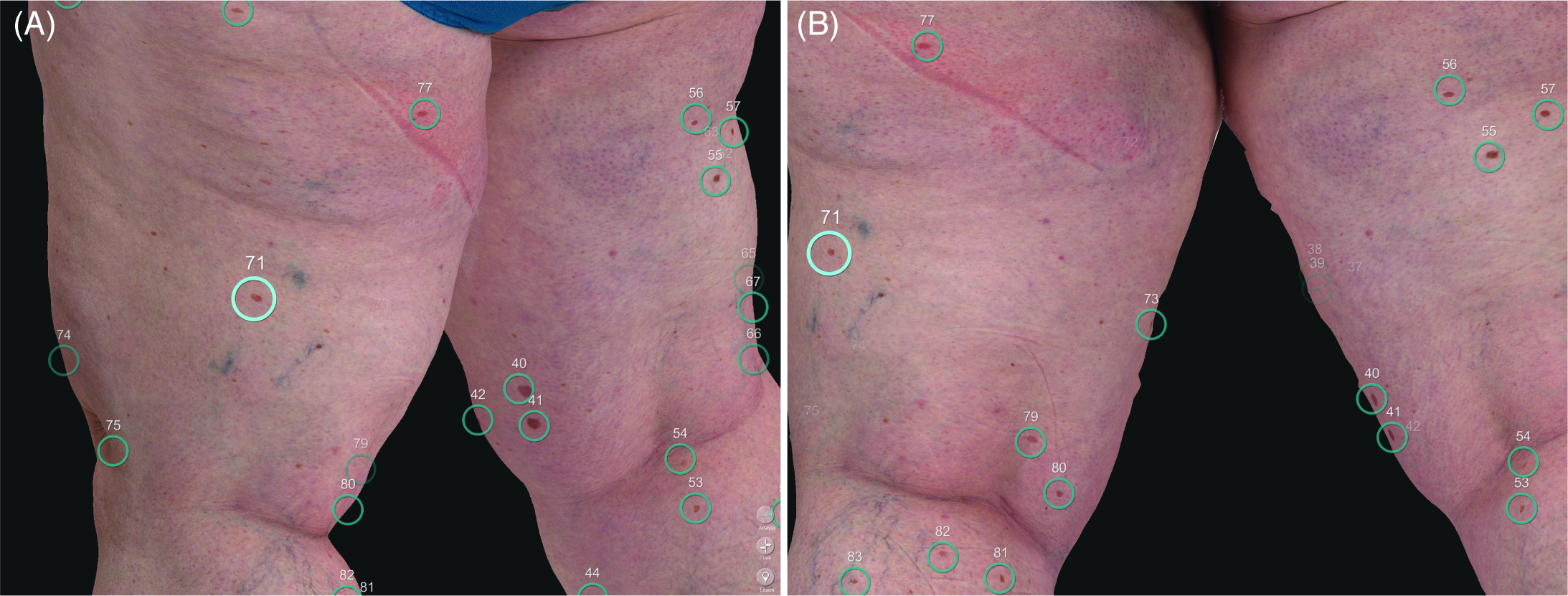

Supplement: Supplementary Figure 1 — (A) The rings without pigmented lesions (#79, #80) alert that there are naevi on the medial aspect of the leg which are not visible on this lateral 2D screen shot (B). In practice, the touch screen allows the body map to be rotated and the images viewed. [file Image_1.TIF]
